# Development and evaluation of extracorporeal membrane oxygenation nursing education program for nursing students using virtual reality

**DOI:** 10.1186/s12909-024-05057-2

**Published:** 2024-01-26

**Authors:** Hanna Lee, Jeong-Won Han, Junhee Park, Soyoon Min, Jihey Park

**Affiliations:** 1 Department of Nursing, Gangneung-Wonju National University, Wonju-si, Gangwon-do Republic of Korea; 2 College of Nursing Science, Kyung Hee University, 26, Kyunghee-daero, Seoul, Dongdaemun-gu 02453 Republic of Korea; 3 College of Nursing Science, Dongnam Health University, Seongnam-si, Gyeonggi-do Republic of Korea; 4 Department of Nursing, Graduate School, Kyung Hee University, Seoul, Republic of Korea

**Keywords:** Clinical reasoning, Education, Nurse, Simulation, Student, Knowledge, Extracorporeal membrane oxygenation

## Abstract

**Background:**

This study aims to improve nursing students’ ability to care for critically ill patients through education in extracorporeal membrane oxygenation (ECMO) nursing.

**Methods:**

This study developed a virtual reality (VR) simulation program for the five-step ECMO nursing of the Analysis, Design, Development, Implement, and Evaluation (ADDIE) model and used an equivalent control group pre-test and post-test no-synchronized design to verify the effect. The participants of this study were fourth-year nursing students enrolled in nursing departments at three universities in Seoul, Gangwon, and Gyeonggi in South Korea; it included 66 participants, 33 in each of the experimental and control groups. The program consisted of pre-training, orientation, VR simulation, and debriefing.

**Results:**

The interaction effect of the intervention and control groups with time points using the ECMO nursing VR simulation program was rejected due to no statistically significant difference in knowledge (F = 1.41, *p* = .251), confidence (F = 1.97, *p* = .144), and clinical reasoning capacity (F = 2.85, *p* = .061). However, learning immersion (t = 3.97, *p* < .001) and learning satisfaction (t = 4.25, *p* < .001) were statistically significantly higher in the experimental group than in the control group.

**Conclusion:**

VR simulation program for ECMO nursing developed in this study is a potential educational method that positively affects the learning immersion and learning satisfaction of nursing students.

## Introduction

ECMO stands for extracorporeal membrane oxygenation. The ECMO machine is similar to the heart-lung by-pass machine used in open-heart surgery [1]. It pumps and oxygenates a patient’s blood outside the body, allowing the heart and lungs to rest. When you are connected to an ECMO, blood flows through tubing to an artificial lung in the machine that adds oxygen and takes out carbon dioxide; then the blood is warmed to body temperature and pumped back into your body [2–4]. ECMO is used not only in acute heart failure, decompensated chronic heart failure, but also in acute severe respiratory failure and CPR situations [5, 6]. ECMO can greatly help in patient treatment by increasing the survival rate of critically ill patients [7]. COVID-19, which occurred in 2020, there was a shortage of health provider and resources in a crisis situation in which the number of critically ill patients approached 1,000. Accordingly, In Korea, ‘Korean clinical practice guideline for ECMO’ were announced [8]. The Korean government has selected nationally designated hospitals for critical care management, expanded intensive care units (ICUs), and trained students to secure dedicated critical care nursing personnel capable of ECMO nursing [9]. However, the supply and demand for nurses who can care for critically ill patients are complicated. Training the personnel is time-consuming; in-depth education and specific training, such as movement management and changing protective clothing, are required for the special circumstances of COVID-19 [10].

During the COVID-19 pandemic, the Extracorporeal Life Support Organization (ELSO) developed ECMO guidelines in the context of COVID-19 for ECMO centers and medical staff worldwide. ELSO recommends that national governments work with the private sector to secure resources during the COVID-19 pandemic and that networks be used at the local level to cooperate with equitable service delivery and staffing in the region [11]. In addition to emphasizing that ECMO training programs and staffing are important at the institutional level, it also advises maintaining a patient-nurse ratio of 1:1 and deploying ECMO specialists [11]. However, there is an insufficient supply of nurses skilled in the care of ECMO patients, and educational programs related to the management of ECMO patients are limited.

To date, ECMO training has been dominated by instructor-centered training methods such as lectures and skills training. However, training using e-learning and high-fidelity simulators has recently been conducted [12–18]. In a previous study targeting nurses [19], a high-fidelity simulation was performed for nurses working in ICUs during a three-day workshop; however, there were limitations in conducting long hours of group education during periods of high COVID-19 transmission.

While COVID-19 has come to an end, we still face the possibility of new infectious diseases emerging. We should shift our focus to proactive learning to forge a better future beyond the shadows of concluded pandemics. We have witnessed how rapidly situations can change during a pandemic, such as the case with COVID-19. Being prepared to adapt to swiftly evolving environments is crucial. Therefore, emphasizing the importance of learning and cultivating the ability to prepare for new infectious diseases is necessary. However, repeatedly educating premedical professionals during a pandemic is exceedingly difficult. Professionals, such as ECMO nurses, cannot be trained in a short time; thus, generating interest and providing knowledge about ECMO nursing to nursing students is necessary. In addition, due to COVID-19, nursing students at many universities have been restricted from practicums in ICUs and have lost opportunities to indirectly experience ECMO nursing. Thus, educational programs are needed to supplement students’ knowledge and experiences. Academics in various fields use virtual reality (VR), a cutting-edge technology that allows humans to interact with computers by making them feel like they are in a virtual space, to educate students [20]. By incorporating these technological characteristics into education, VR simulations create a VR space that mimics the nursing practice scenario and allows students to make situational clinical decisions and provide nursing care accordingly [21]. VR simulations enable students to experience real patient care vicariously in a safe environment without space constraints, thereby enabling repetitive practice and providing immediate feedback, especially during a pandemic [20]. Thus, this study aimed to develop an ECMO nursing education program for nursing students who are pre-medical professionals and evaluate its effects on education.

### Study hypotheses

#### Hypothesis 1

There are differences in the knowledge of ECMO nursing between the intervention group applying the ECMO nursing VR simulation program and the control group.

#### Hypothesis 2

There are differences in ECMO nursing-related confidence between the intervention group applying the ECMO nursing VR simulation program and the control group.

#### Hypothesis 3

There are differences in ECMO nursing-related clinical reasoning capacity between the intervention group applying the ECMO nursing VR simulation program and the control group.

#### Hypothesis 4

There are differences in learning immersion between the intervention group applying the ECMO nursing VR simulation program and the control group.

#### Hypothesis 5

There are differences in learning satisfaction between the intervention group using the ECMO nursing VR simulation program and the control group.

## Methods

### Study design and setting

In this study, a VR simulation program for ECMO nursing was developed. We evaluated its effects on ECMO nursing knowledge, confidence, clinical reasoning capacity, learning immersion, and learning satisfaction in fourth-year nursing students using an equivalent control group pre- and post-test design.

### Participants

This study was conducted among fourth-year nursing students from three universities in Seoul, Gangwon, and Gyeonggi, Korea. The selection criteria for participants in this study were fourth-year nursing students, who had never received ECMO education through VR, and who consented to this study. The exclusion criteria excludes participants who are at high risk of developing health problems due to the use of Oculus products used in this study. In this study, the minimum number of samples for the implementation stage to test program effectiveness was determined to be 26 participants per group with a 1:1 assignment, a test power of 0.80, a significance level of 0.05, and an effect size of 0.80 when using a two-tail test of the difference between two independent means (two groups) in the G*Power 3.1.2 program. The calculation of the sample size was based on the effect size of learning satisfaction (effect size: d = 1.33), measured as the main variable in a previous study [22] that applied a VR simulation program to nursing students based on learning satisfaction, which was also one of the main variables of this study. This study set the effect size (f) as 0.80 (large) and included 66 participants as study subjects, 33 per group, considering a dropout rate of 20.0% given the social distancing due to COVID-19. To recruit participants, recruitment notices including research and program details were posted at three universities. Students who voluntarily expressed interest in participating in the research were encouraged to contact the researchers directly after reviewing the recruitment announcements within their respective campuses. Subsequently, trained research team members individually explained the purpose and procedures of the research, obtaining voluntary consent from interested participants. Prior to the study, researchers obtained informed consent from participants who agreed to take part. Using the assignment results generated by the http://randomization.com website, the experimental and control groups were established. In this study, a 1:1 allocation ratio was employed, with 33 participants assigned to both the experimental and control groups. The randomization process was conducted with block sizes of 2 and 4 units concurrently. The random permutations generated by the http://randomization.com website were implemented using Stata 9.0 (StataCorp, College Station, TX). Participants were categorized into Group A (experimental) and Group B (control) according to the assignment results. Participants were recruited from three schools with 22, 24, and 20 individuals, respectively. To mitigate the spread of the experiment’s effects, student experiments were scheduled without overlapping, and participants were advised against disclosing insights gained from the experiment.

### Program development

The program was developed according to the five-step analysis, design, development, implementation, and evaluation (ADDIE) model, which was used to develop the teaching and learning methods [23].

#### Analysis stage

The analysis stage involved obtaining the basic data necessary to develop the VR simulation scenarios and educational programs. Here, educational needs were investigated through a literature review of ECMO nursing-related simulations and interviews with three nurses with experience working in ICUs, two nursing professors, and two fourth-year nursing students. Data searches were performed using eight databases. The databases for foreign literature searches included PUBMED, Scopus, ProQuest, and CINAHL, whereas DBpia, KISS, National Assembly Library, and Korea Education and Research Information Service were used for domestic literature searches. In the literature review and interviews, we identified what nursing students should learn about ECMO through VR, the operating hours of VR programs, and the content necessary for pre-training and debriefing configuration.

#### Design stage

At this stage, the learning goal was set based on the analysis results, and the study design and operational methods suitable for achieving the goal were determined. In this study, an ECMO nursing program was developed based on the case of a critically ill patient diagnosed with ARDS as a COVID-19 complication. The important nursing content selected during the analysis stage was set as the scenario and algorithm for the VR program.

#### Development stage

In the development stage, algorithms and scenarios related to nursing care were developed for ECMO patients, and a pre-learning curriculum was constructed to help them acquire relevant knowledge before performing the VR simulation. To review the VR simulation algorithm, the scenario and prior learning compositions, and the suitability and applicability of the learning objectives, opinions were collected from three nurses with experience working in ICUs and two nursing professors. The Content Validity Index [CVI] for experts was checked. The Item Content Validity Index (I-CVI) for each item was found to be 1.0, indicating unanimous agreement among the expert panel that all items were relevant and appropriate for the intended construct. Before applying the developed program to the participants, its potential as a nursing education program was examined by simulating it with one clinical nurse, and any problems were corrected. A preliminary investigation was also conducted to determine whether there was any difficulty in understanding the educational content and whether additional educational content was require. The final VR simulation education program was tested by two fourth-year nursing students and one ICU nurse to increase research fidelity.

#### Implementation stage

In the implementation stage, the developed program is applied to both the experimental and control groups. The control group underwent pre-learning, while the experimental group followed the sequence of pre-learning, orientation, simulation running, and debriefing.

#### Evaluation stage

A pre-test was conducted with the experimental and control groups on their general characteristics, knowledge of ECMO nursing, confidence, and clinical reasoning capabilities before the VR program and lectures. A post-test was also performed with both groups regarding knowledge of ECMO nursing, confidence, clinical reasoning capability, learning immersion, and learning satisfaction after the VR program and lectures. Participants were subjected to a second post-test survey after two weeks.

### ECMO VR nursing simulation program

**Fig. 1:**
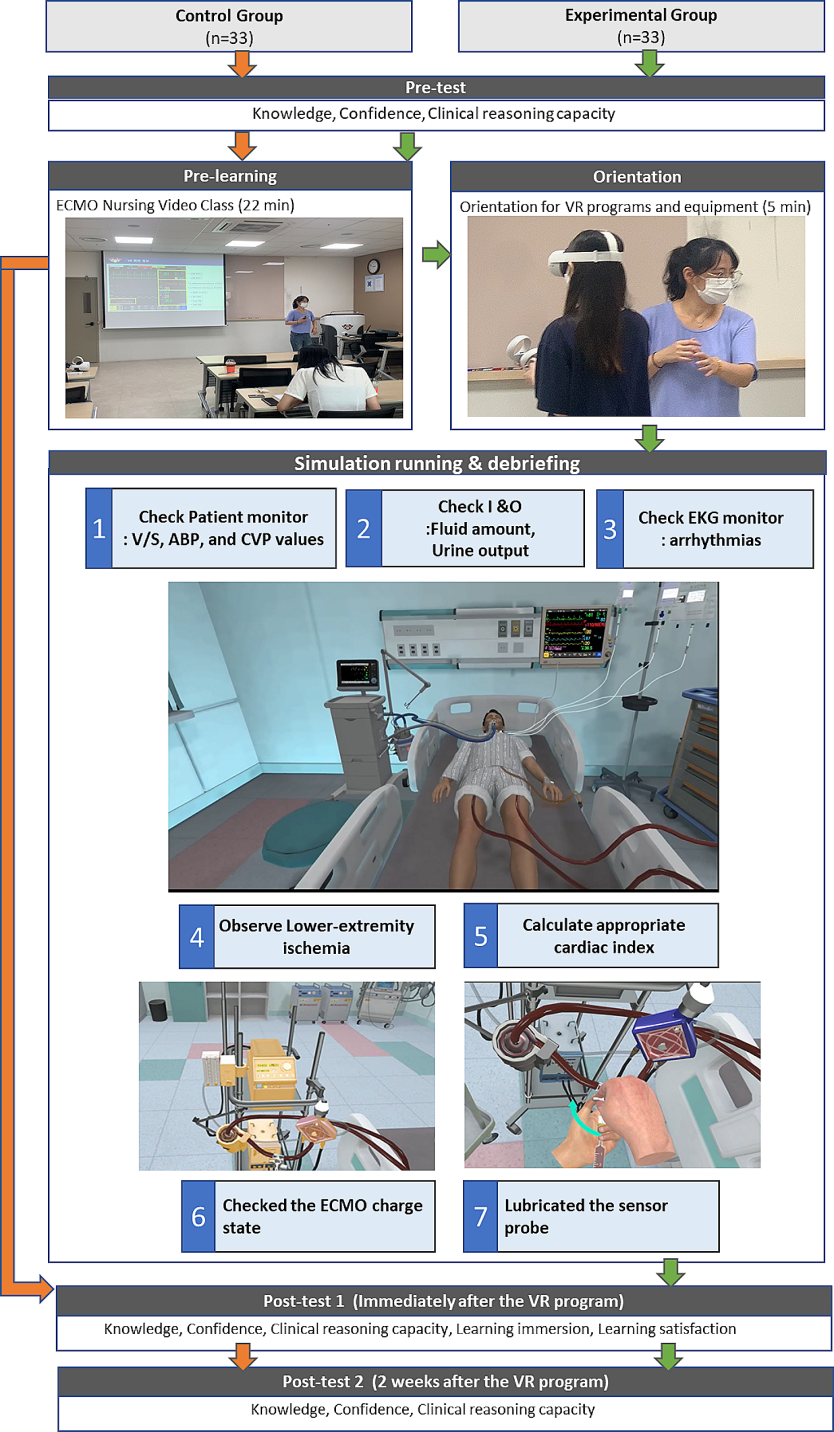
ECMO VR Nursing simulation program

The ECMO VR Nursing simulation program consists of pre-class, orientation, simulation running, and debriefing stages (Fig. 1).

#### Pre-learning

The pre-learning session has a duration of 22 min, covering various learning contents related to ECMO. These include the definition, types, indications of ECMO, the structure of the ECMO machine, ECMO priming & cannulation, and nursing care during ECMO initiation, maintenance, and weaning. Considering the potential variation in content delivery due to instructors, time constraints, and individual student needs, a pre-recorded lecture was utilized. This approach ensured that both the control group and the experimental group had access to identical learning material.

#### Orientation

Before the VR experience, a trained research assistant provided an orientation on the VR program and equipment to a participant in the experimental group. Subsequently, the simulation was initiated. During the VR simulation, the participant executed the procedure using an Head-Mounted Display (HMD) and controller (Oculus). The orientation scene of the equipment is depicted in Fig. 1. Researchers also briefed the participant on a precaution file related to the use of Oculus products. The participant placed the HMD on their head and performed head movements in various directions (up, down, left, and right) to experience a 360-degree virtual reality ward through the display in front of them. Using controllers held in both hands, the participant engaged with the content in a sequential order to acquir83e the necessary skills.

#### Simulation running

The duration of the program was slightly different for each student and took a minimum of 5 min to a maximum of 15 min. The students performed VR programs while wearing VR headsets and controllers. The virtual patient was admitted to the ICU on the second day after the diagnosis of ARDS. Hypoxemia (PO_2_ 47.0∼41.7mmHg) persisted despite mechanical ventilation control, and a venous-vein extracorporeal membrane oxidizer is being applied to improve lung oxygenation. For the virtual patient to maintain ECMO, mechanical ventilation was in assist-controlled mode, pressure-controlled mode (20-25cmH2O), tidal volume 500mL, respiration rate 12 times per minute, inhaled oxygen concentration 0.5, and positive end-expiratory pressure (5cmH2O) was maintained. Students experience various nursing interventions sequentially in the ICU. Students look at the patient monitor, check the Vital Signs, Arterial Blood Pressure, and Central Venous Pressure values, and record them on the Electronic Medical Record. The balance of intake and output was also checked, and the amount of fluid injected for 1 h and the amount of urine excreted for 1 h were checked and recorded in the EMR. The students observed the patient’s EKG monitor and checked for arrhythmias. In addition, students will observe lower-extremity ischemia on the side of the femoral artery into which the ECMO cannula is inserted. Students used the calculator to calculate whether the appropriate cardiac index was maintained according to ECMO flow, checked the ECMO charge state, and lubricated the sensor probe in the event of an alarm.

#### Debriefing

For the debriefing of the experimental group, a debriefing plan based on the scenario algorithm was prepared and utilized to assess the expected nursing performance from learners after the VR experience. During debriefing, participants are prompted with questions such as ‘What went well and what was lacking? Why do you think that?’ and ‘What do you think was the most difficult and embarrassing thing for you, and why?

### Measurement

#### Knowledge of ECMO nursing

In this study, the researchers developed a tool comprising 15 questions based on the literature to assess their knowledge of ECMO nursing. Two nursing professors with experience in teaching critical care nursing courses or working in ICUs and three nurses with more than five years of ICU experience validated it. Each question was scored 0 points for a wrong answer and 1 point for a correct answer, with higher scores indicating higher degrees of ECMO nursing knowledge.

#### Confidence in ECMO nursing

Confidence in ECMO nursing was evaluated on a five-point scale using the two questions proposed by Thomas et al. [24], in which higher scores indicated higher degrees of confidence in ECMO nursing.

#### Clinical reasoning capability

This study used an evaluation tool developed by Liou et al. [25] for clinical reasoning. The Korean version was verified by Han and Jeong [26] and comprised 15 questions (measured on a 5-point scale), with higher scores indicating higher degrees of clinical reasoning capacity. In the study by Liou et al. [25], the reliability of the tool was found to be Cronbach’s α = 0.94, whereas the study by Han and Jeong [26] showed Cronbach’s α = 0.93. Cronbach’s α in this study was 0.97.

#### Learning immersion

To measure nursing students’ immersion in learning, we used a 10-question, 5-point Likert flow short scale developed by Engeser and Rheinberg [27] and validated by Yoo [28] through translation and reverse translation into Korean. Higher scores indicate higher levels of immersion in learning. Cronbach’s α was 0.92 at the time of development [27], and 0.84 in Yoo’s [28] study. Cronbach’s α in this study was 0.92.

#### Learning satisfaction

In this study, learning satisfaction was measured using a numeral rating scale (NRS) ranging from 0 = “very dissatisfied to 10 = “very satisfied.” Higher scores indicate higher degrees of satisfaction with learning.

### Data collection and analysis

Considering the COVID-19 situation, the research team members trained in daily quarantine met one-on-one with the study participants to explain the purpose and method of the research and conduct the study. Data were analyzed using SPSS for WIN/23.0 (SPSS Data Solution. co), and the Shapiro-Wilk test was performed to test for normality of variables before program application. Participants’ general characteristics were analyzed and pre-homogeneity tests for variables were performed using the chi-square, Fisher’s exact, and independent t-tests. After the intervention, a two-way repeated-measures analysis of variance (ANOVA) was performed to determine the effect on the variables, including ECMO nursing knowledge, confidence, and clinical reasoning capability in the experimental and control groups. Learning immersion and satisfaction were assessed using independent t tests.

## Results

### Homogeneity test of subjects for general characteristics

The majority of the participants 55 (93.3%) were female, with an average age of 23.3 ± 4.3 years. Fourteen (21.2%) participants had a religion, whereas 52 (78.8%) did not. Regarding satisfaction with the clinical practicum, 46 participants (69.7%) were “satisfied,” 18 (27.3%) reported “average” satisfaction and 2 (3.0%) were “dissatisfied.” Regarding satisfaction with their majors, 45 (68.2%) participants were “satisfied” and 21 (31.8%) considered themselves “below average.” As for their satisfaction with school life, 45 participants (68.2%) were “satisfied” and 21 (31.8%) reported “below average” satisfaction. Regarding experience with ECMO education, 61 participants (92.4%) had experience, whereas five (7.6%) had none. Students with ECMO training experience were surveyed regarding their training methods, and the following results were found: 46 (74.2%) had taken a lecture, 14 (22.6%) participated in a clinical practicum, and two (3.2%) performed a simulation. No factors showed statistically significant differences in the homogeneity test between the intervention and control groups according to their general characteristics (Table 1).

**Table 1:** Homogeneous test of general characteristics of the participants (N = 66)

Variables	Category	Total (*n* = 66)	Control (*n* = 33)	Experimental (*n* = 33)	χ^2^ or t	p
		n(%) or M ± SD	n(%) or M ± SD	n(%) or M ± SD		
Gender	Male	11(16.7)	6 (9.1)	5 (7.6)	0.11	0.741
	Female	55(93.3)	27 (40.9)	28(42.4)		
Age (yr)		23.3 ± 4.3	23.3 ± 5.1	23.3 ± 3.3	− 0.021	0.983
Religion	Yes	14(21.2)	6 (9.1)	8 (12.1)	0.363	0.547
	None	52(78.8)	27 (40.9)	25 (37.9)		
Satisfaction of clinical practice	Satisfied	46(69.7)	24 (36.4)	22 (33.3)	1.74^†^	0.575^†^
	Moderate	18(27.3)	9 (13.6)	9 (13.6)		
	Dissatisfied	2(3.0)	0 (0.0)	2 (3.0)		
Satisfaction of major	Satisfied	45(68.2)	22 (33.3)	23 (34.8)	0.07	0.792
	Below Moderate	21(31.8)	11 (16.7)	10 (15.2)		
Satisfaction of college	Satisfied	45(68.2)	22 (33.3)	23 (34.8)	0.07	0.792
	Below Moderate	21(31.8)	11 (16.7)	10 (15.2)		
ECMO education experience	Yes	61(92.4)	30 (45.5)	31 (47.0)	0.22	1.000^†^
	None	5(7.6)	3 (4.5)	2 (3.0)		
Learning method (*n* = 61)	Lecture	46(74.2)	24 (38.7)	22 (35.5)	0.63	0.879^†^
	Clinical practice	14(22.6)	6 (9.7)	8 (12.9)		
	Simulation practice	2(3.2)	1 (1.6)	1 (1.6)		

M = Mean, SD = Standard deviation

### Tests for normality and homogeneity of the pre-dependent variables

In this study, the pre-dependent variables, including knowledge and confidence in ECMO nursing and clinical reasoning capacity, secured normality and homogeneity in both groups (Table 2).

**Table 2:** Homogeneous test of dependent variable (*N* = 66)

Variables	Control (*n* = 33)	Experimental (*n* = 33)	t (*p*)
	M ± SD	M ± SD	
Knowledge	11.82 ± 1.530	11.12 ± 2.62	-1.32(0.192)
Confidence	6.94 ± 3.98	6.30 ± 4.51	− 0.607(0.546)
Clinical reasoning capacity	35.97 ± 10.81	33.88 ± 12.97	− 0.711(0.479)

M = Mean, SD = Standard deviation

### Verification of the effectiveness of the VR Simulation Program for ECMO nursing

#### Knowledge of ECMO nursing

Hypothesis 1, which suggested that there would differences in the knowledge of ECMO nursing between the intervention group applying the ECMO nursing VR simulation program and the control group, was tested. The main effect found between the time points was that both the experimental (F = 34.81, *p* <.001) and control groups (F = 29.47, *p* <.001) significantly increased their knowledge of ECMO nursing. However, there was no statistically significant difference between the pre-test (F = -1.320, *p* = .192), Posttest 1 (F = 0.723, *p* = .472), and the second post-test (F = 0.107, *p* = .915) in both the experimental and control groups. The effect of the interaction between the group and time point (F = 1.41, *p* = .251) was also not statistically significant; thus, the first hypothesis was rejected (Table 3).

**Table 3:** Effect of VR simulation program (*N* = 66)

Variables	Experimental (*n* = 33)	Control (*n* = 33)	t (*p* value^b^)	F (*p* value^c^) for interaction
	Mean ± SD	Mean ± SD		
***Knowledge***				
Pre-test	11.12 ± 2.619	11.82 ± 1.530	-1.320 (0.192)	1.41(0.251)
Post-test 1	13.73 ± 1.232	13.48 ± 1.482	0.723 (0.472)	
Post-test 2	14.12 ± 1.219	14.09 ± 1.071	0.107 (0.915)	
F (*p* value^a)^	34.81 (< 0.001)	29.47 (< 0.001)		
***Confidence***				
Pre-test	6.30 ± 4.510	6.94 ± 3.984	− 0.607 (0.546)	1.97(0.144)
Post-test 1	13.27 ± 4.785	12.52 ± 3.063	0.766 (0.446)	
Post-test 2	13.61 ± 3.791	12.61 ± 3.041	1.182 (0.242)	
F (*p* value^a)^	65.48 (< 0.001)	60.56 (< 0.001)		
***Clinical reasoning capacity***				
Pre-test	33.88 ± 12.971	35.97 ± 10.812	− 0.711 (0.479)	2.85(0.061)
Post-test 1	52.61 ± 12.296	50.45 ± 9.394	0.799 (0.427)	
Post-test 2	53.39 ± 12.865	50.70 ± 9.026	0.986 (0.328)	
F (*p* value^a)^	91.67 (< 0.001)	66.03 (< 0.001)		
***Learning immersion***				
Post-test 1	43.39 ± 5.836	37.82 ± 5.576	3.968 (< 0.001)	N/A
***Learning satisfaction***				
Post-test 1	9.06 ± 1.088	7.55 ± 1.734	4.252 (< 0.001)	N/A

*p* value^a^ for within group comparisons were computed using repeated measures ANOVA

*p* value^b^ for between group comparisons were computed using independent t-test

*p* value^c^ for between group comparisons at baseline were computed using t-way repeated measures ANOVA

N/A: not applicable

#### Confidence in ECMO nursing

Hypothesis 2, which proposed that there would be differences in the confidence in ECMO nursing between the intervention group applying the ECMO nursing VR simulation program and the control group, was tested. The main effect identified between the time points was that both the experimental (F = 65.48, *p* <.001) and control groups (F = 60.56, *p* <.001) significantly increased their confidence in ECMO nursing. However, there was no statistically significant difference in the main effect among the time points (pre-test: F = − 0.607, *p* = .546; post-test 1: F = 0.766, *p* = .446; and post-test 2: F = 1.182, *p* = .242) in either the experimental or control groups. The effect of the interaction between group and time point (F = 1.97, *p* = .144) was also not statistically significant; thus, the second hypothesis was rejected (Table 3).

#### Clinical reasoning capability

Hypothesis 3, which theorized that there would be differences in ECMO nursing-related clinical reasoning capacity between the intervention group applying the ECMO nursing VR simulation program and the control group, was tested. The main effect identified between the time points was that both the experimental (F = 91.67, *p* <.001) and control groups (F = 66.03, *p* <.001) significantly increased their clinical reasoning capability in ECMO nursing. However, there was no statistically significant difference in the main effect among the time points (pre-test: F = − 0.711, *p* = .479; post-test 1: F = 0.799, *p* = .427; and post-test 2: F = 0.986, *p* = .328) in both the experimental and control groups. The effect of the interaction between the group and time point (F = 2.85, *p* = .061) was also not statistically significant; thus, the third hypothesis was rejected (Table 3).

#### Learning immersion

Hypothesis 4, which posited that there would be differences in learning immersion between the intervention group applying the ECMO nursing VR simulation program and the control group, was tested. The learning immersion of the experimental group was found to be statistically significantly higher than that of the control group (t = 3.97, *p* <.001); thus, the fourth hypothesis was accepted.

#### Learning satisfaction

Hypothesis 5, which proposed that there would be differences in learning satisfaction between the intervention group applying the ECMO nursing VR simulation program and the control group, was tested. The learning immersion of the experimental group was found to be statistically significantly higher than that of the control group (t = 4.25, *p* <.001); thus, the fifth hypothesis was accepted.

## Discussion

This study aimed to improve nursing students’ ability to care for critically ill patients through ECMO nursing VR learning in a pandemic situation resulting from infectious diseases such as COVID-19. The implications of the program’s effectiveness are as follows.

First, the hypothesis that “there are differences in the knowledge of ECMO nursing between the intervention group applying the ECMO nursing VR simulation program and the control group” was rejected. This result is similar to that of a study [29] in which four hospitals in the United States implemented a screen-based pediatric ECMO simulation training program for medical professionals. Regarding knowledge of mechanical ventilation nursing in the intervention group, which applied the mechanical ventilation nursing simulation program using VR in Korea, and the control group, there was no significant difference in the interaction effect between the group and time point [30]. However, the results were inconsistent with those of a study [31] in Taiwan, which showed that the experimental group had higher knowledge than that of the control group after providing nursing students with educational materials related to chemotherapy administration using VR. Thus, the effect of knowledge is not consistent with that of simulation practice. It seems that the subjects tended to consider VR simulations an opportunity to apply and combine existing knowledge rather than increasing their knowledge by operating it for a long time. Some have speculated that there was no significant difference as this study also measured the basic knowledge of ECMO related to the scenario rather than newly learned specific knowledge. As simulation-based education is based on the integrated use of knowledge while participating in the process of solving problems in a given situation, there seems to be a limitation in measuring the effectiveness of education by measuring knowledge through simple questions. In addition, the knowledge measurement tool in this study was intended to measure whether participants had correct or incorrect knowledge; thus, it might have been unable to detect significant differences in the knowledge level between the experimental and control groups. Most of the students in this study reported that they had ‘previously received ECMO education.’ Therefore, the impact of the current education may not be readily apparent as they had already been exposed to ECMO-related education. Several previous studies that trained medical staff on ECMO simulations and evaluated their performance have shown the simulations’ effects on performance [13–15, 17]. Thus, future studies should verify the effectiveness of ECMO nursing VR simulation programs using tools to evaluate empirical knowledge and nursing performance skills. Future research will need to validate this training program among students without prior experience in ECMO training. Furthermore, future studies should verify the effectiveness of ECMO nursing VR simulation programs using tools to evaluate empirical knowledge and nursing performance skills.

Second, the hypothesis, “There are differences in ECMO nursing-related confidence between the intervention group applying the ECMO nursing VR simulation program and the control group” was rejected. This result is similar to that of a study [29] that applied a screen-based pediatric ECMO simulation training program, in which there was no statistically significant difference in self-efficacy. A study [32] that developed a high-fidelity ECMO simulation in the UK reported that it allowed students to learn skills in an interactive environment without harming real patients and boosted their confidence. However, an analysis of the confidence score data in this study revealed that the mean confidence of the experimental group increased from 6.30 in the pre-test to 13.61 in the post-test, while the control group’s confidence increased from 6.94 to 12.61. However, although the difference in confidence increase in the experimental group appeared to be greater than that of the control group, it was not statistically significant. These results suggest that because ECMO is a difficult topic to understand in a short time period [33], it is difficult to improve confidence with a single training session, as shown in this study. However, the ECMO VR simulation training developed in this study can improve students’ confidence because it allows for the safe and planned implementation of ECMO-related training [32]. Further studies are required to evaluate the improvement in confidence through iterative applications of the program.

Third, the hypothesis that “There are differences in ECMO nursing-related clinical reasoning capacity between the intervention group applying the ECMO nursing VR simulation program and the control group” was rejected. This is contrary to the results of a study in Korea, in which a simulation program using VR for nursing students was effective in improving students’ clinical reasoning capabilities [30]. Clinical reasoning capability is the thought process for gathering and analyzing patient information, assessing the importance of the analyzed information, and determining alternative behaviors [34]. Simulation-based training is expected to enhance clinical reasoning capabilities because it provides an opportunity for nursing students and nurses to develop and maintain technical proficiency in high-risk, rare events without fear of harming patients [32]. Nonetheless, clinical reasoning capability did not improve over a short period, which seems to explain why there was no significant difference between the experimental and control groups. However, previous studies [35, 36] have suggested that a program using various simulation methods to educate medical staff on all aspects related to ECMO is needed to improve nursing students’ ability to treat patients on ECMO. Likewise, repeated practice of various clinical situations using the ECMO nursing VR simulation program developed in this study would help improve their clinical reasoning capacity.

Fourth, the hypothesis that “There are differences in learning immersion between the intervention group applying the ECMO nursing VR simulation program and the control group” was accepted, which is consistent with the results of a previous study [37] that the motivation to learn increased after applying the education program using VR. Due to the current COVID-19 pandemic, the demand for ECMO is unprecedented, and its management is highly complex; however, nursing students have found it difficult to acquire relevant skills [38]. The ECMO nursing VR simulation program developed in this study extends from traditional instructor-led learning methods to real-world clinical experiences that are rarely encountered. We believe that such experiences can be an effective way to improve nursing students’ learning immersion. In particular, the VR in this study used an HMD to seal the user’s audiovisual and other senses and used visual elements in the ICU and auditory elements, such as the patient’s breathing sounds and alarms, to create a lifelike experience that ensured students’ learning immersion. Therefore, the provision of various teaching methods, including VR, to assist nursing students in their education and to find ways to improve their immersion in learning is necessary.

Fifth, the hypothesis that “There are differences in learning satisfaction between the intervention group applying the ECMO nursing VR simulation program and the control group” was accepted. This is consistent with results of a previous study [30] in which the experimental group had higher learning satisfaction than the control group in a mechanically ventilated nursing VR simulation program for Korean nursing students [30]. Similarly, 95.2% of participants were satisfied with an high fidelity simulation ECMO nursing training program for ICU nurses in France [19]. This was because the educational program developed in this study considered participants’ learning needs and attempted to maximize the educational elements by realistically implementing scenarios and clinical situations with clinical and educational validity in VR. In addition, this result seems to be due to the characteristics of the learning method because immersive VR simulation employs digital devices to learn in a situation completely different from reality, making it highly immersive and distinct from reality, which might have improved students’ learning interest and confidence [39]. High satisfaction with simulation education can improve learners’ internal motivation and, consequently, their clinical performance [40]. Thus, the program developed in this study is expected to enhance students’ competence. A limitation of the study is that it is difficult to generalize since the study was conducted on nursing students at some universities. Therefore, it is recommended to attempt a repeat study to supplement these points in the future. Additionally, research is needed to measure the effectiveness of simulation education that combines various types of simulation modalities.

## Conclusion

ECMO is a challenging issue in nursing education owing to the complex, high-risk, and low-frequency clinical activities that require dynamic decisions. If ECMO education is implemented with VR simulation education to provide HPS-based simulation, students can learn in a safe, clinical environment in a virtual space without being affected by time and place as they are in lecture-based learning methods. The results are expected to be more effective if the ECMO nursing VR simulation program developed in this study is combined with classroom lectures and HPS simulations. However, this study applied the VR simulation training program to students only once, and the learning objective of ECMO could not be acquired in a short period. The advantage of the VR simulation program is that it allows for repeated practice; therefore, future studies should investigate the effect of an increased number of simulation training sessions and its mid- to long-term intervention effects using a longitudinal study design.

ECMO education is a challenging issue in nursing education because of its unique nature of complex, high-risk, and low-frequency clinical activities that require dynamic decisions. If ECMO education is implemented with VR simulation education, students can learn in a safe clinical environment implemented in a virtual space compared to lecture-based learning methods, without being affected by time and place compared to HPS-based simulation. Therefore, the result is expected to be more effective if the ECMO nursing VR simulation program developed in this study is used together with the lecture in the classroom and HPS simulation. However, this study applied the VR simulation training program to the students only once, while the learning object of ECMO cannot be acquired in a short time period. The advantage of the VR simulation program is that it allows repeated practices; so, a future study should investigate the effect of an increased number of simulation training and its mid- to long-term intervention effects in a longitudinal study design.

## Data Availability

The datasets used and analyzed during the current study are available from the corresponding author on request.

## Abbreviations

ADDIE: Analysis, design, development, implement, and evaluation
COVID-19: Coronavirus Disease of 2019
ECLS: Extracorporeal life support
ECMO: Extracorporeal membrane oxygenation
VR: Virtual reality
